# Multiscale Trend Analysis for Pampa Grasslands Using Ground Data and Vegetation Sensor Imagery

**DOI:** 10.3390/s150717666

**Published:** 2015-07-21

**Authors:** Fernando C. Scottá, Eliana L. da Fonseca

**Affiliations:** Department of Geography, Federal University of Rio Grande do Sul, Porto Alegre 91501-970, RS, Brazil; E-Mail: eliana.fonseca@ufrgs.br

**Keywords:** biomass, scale, climate analysis, NDVI

## Abstract

This study aimed to evaluate changes in the aboveground net primary productivity (ANPP) of grasslands in the Pampa biome by using experimental plots and changes in the spectral responses of similar vegetation communities obtained by remote sensing and to compare both datasets with meteorological variations to validate the transition scales of the datasets. Two different geographic scales were considered in this study. At the local scale, an analysis of the climate and its direct influences on grassland ANPP was performed using data from a long-term experiment. At the regional scale, the influences of climate on the grassland reflectance patterns were determined using vegetation sensor imagery data. Overall, the monthly variations of vegetation canopy growth analysed using environmental changes (air temperature, total rainfall and total evapotranspiration) were similar. The results from the ANPP data and the NDVI data showed the that variations in grassland growth were similar and independent of the analysis scale, which indicated that local data and the relationships of local data with climate can be considered at the regional scale in the Pampa biome by using remote sensing.

## 1. Introduction

**Figure 1:**
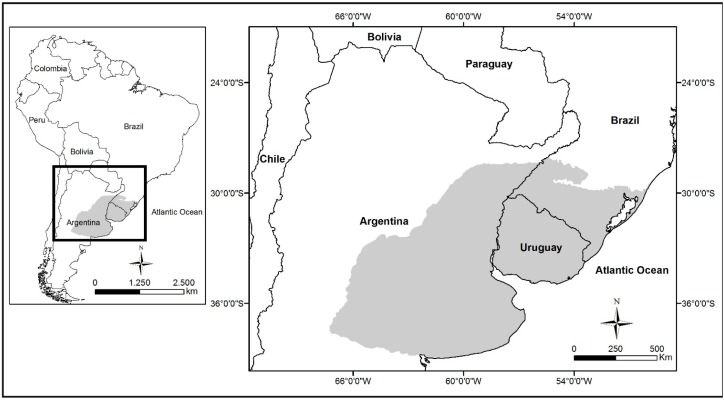
Location of the Pampa biome in Argentina, Uruguay and Brazil (grey-shaded area).

The Pampa biome is located in Argentina, Uruguay and in the southern part of Brazil (Figure 1). This biome is broadly classified as Rio da Prata grassland [1,2], consists of large areas dominated by herbaceous vegetation (primarily grasses) and shrubs [3] and covers an area of approximately 700,000 km^2^ [2]. Furthermore, this biome exhibits extensive biodiversity, with approximately 2200 vegetal species [4]. In this biome, C_3_ and C_4_ species [3] that are adapted to subtropical to temperate climates coexist. Furthermore, this biome serves as a habitat for native and migrant birds [5], especially for neotropical [6] and neartic birds [7] during the winter in the Northern hemisphere. The primary economic activity in this biome is livestock production, and the net primary productivity of the natural grasslands serves as a food source for cattle and sheep. According to the Brazilian Statistics Office (IBGE), 13 million cattle and 5 million sheep reside in the Brazilian portion of the biome [8], which represents 6.8 and 22.6% of the total number of cattle and sheep in Brazil, respectively [9].

Field measurements of aboveground net primary production (ANPP) are generally performed across small fractions of the Earth’s surface at the local scale. Due to this limited measurement scale, environmental studies conducted at regional and global scales must somehow extend field measurements to the appropriate spatial domain [10]. Because of the difficulties that are encountered when working at the regional scale, few researchers have described such research in a single document [11]. Thus, it is not only difficult to access regional scale data but also to understand the entire model system. According to Bettolli *et al.* [12], the lack of extensive records on vegetation growth in Uruguay make it difficult to analyse climate variability and its impacts on the productivity of this biome.

Satellites for obtaining remote sensing data from Earth provide temporal and spatial information of the biosphere that can be used to assess the impacts of environmental changes on terrestrial ecosystems [13,14]. Electro-optical sensors are commonly used for monitoring vegetation. These sensors generate multispectral images from the energy reflected by targets located on the Earth’s surface in specific wavelength bands of the electromagnetic spectrum, primarily in the red and near-infrared region, where vegetation interacts in a characteristic pattern with the incident solar radiation [15]. The variations in the reflectance of an individual leaf can be used to indicate the physiological conditions of a plant [16]. The region of the electromagnetic spectrum between 0.4 and 0.7 µm is referred to as photosynthetic active radiation (PAR) and is used by plants in the physiological processes of biomass accumulation, which justifies the large absorption and low reflectance presented by plants in this wavelength range [17,18]. Between 0.7 and 1.3 µm (near-infrared-NIR), greater reflectance and transmission of incident radiation occurs because this radiation is not used in plant growth processes [19]. The scattering of radiation in this portion of the electromagnetic spectrum is used as a cooling process in the leaf and prevents the accumulation of energy within the leaf [15]. Above 1.3 µm, the incident radiation is partitioned as a function of the absorption bands of water in the mesophyll at wavelengths of 1.4, 1.9, and 2.7 µm [19].

The morphological and physiological changes in the canopy promote changes in the absorbed, transmitted and reflected electromagnetic radiation fractions [20,21]. These variations can be analysed and quantified to monitor vegetation using orbital remote sensing [22]. Fisher *et al.* [23] indicated that it is possible to scale up data collected in the field regarding the physiology of green leaves in studies of satellite images. The vegetation phenology was fit to a sigmoidal growth model using data from Landsat 5 and Landsat 7 and consistently represented the spatial and temporal variations of deciduous forests in Connecticut, Rhode Island and Massachusetts in the USA. An inverse relationship was found between the increase in plant biomass and the energy reflected in the red portion of the electromagnetic spectrum (0.6 to 0.7 µm), as described by Curran and Milton [24] and Goel [22]. The increased energy absorption in this range of the electromagnetic spectrum directly results from greater amounts of photosynthetic pigments [25,26,27,28]. From 0.7 to 1.3 µm, the amount of reflected energy increases as the amount of biomass increases due to increases in the amount of intercellular space with terrain area. This increase promotes an increase in the successive reflections and refractions of electromagnetic radiation within the various layers of the leaf mesophyll. Tucker [29] showed that the spectral range between 1.55 and 1.75 μm is suitable for monitoring the presence of water in a canopy and that shortwave infrared band (1.3–2.5 μm) information can be used to obtain the moisture contents of plant canopies. From wavelengths of 1.3 µm, the reflectance increases in the dry vegetation due to drought and natural senescence because the vegetation reflectance is modulated by the absorption bands of water in this portion of the electromagnetic spectrum [27,30,31]. Carter [32] showed that the reflectance between 0.4 and 0.7 µm (visible) is more sensitive to leaf stress than the region between 0.76 and 2.5 μm (NIR) and that the NIR region is a consistent indicator when stress is severe and leaf dehydration occurs.

Mathematically combining the reflectance of two or more bands, known as vegetation indices, can be performed to provide information regarding the biophysical properties of vegetation [33,34,35,36]. Vegetation indices have several advantages because they condense the volume of data to be analysed [37] and allow one to quantify and assess differences in growing conditions. In addition, these indices are related to the biophysical variables and structural and physiological features of vegetation [38,39]. One of the most commonly used indices is the Normalized Difference Vegetation Index (NDVI; [NDVI = (Ρir − ρR) / (ρIR + ρR)]), which was proposed by Rouse *et al.* [40]. In the NDVI, ρIR is the reflectance in the near-infrared region and ρR is the reflectance in the red region. The NDVI is related to biophysical vegetation properties, such as leaf area index (LAI), various vegetation conditions and the amount of biomass [41]. Thus, the NDVI can be used to monitor the ANPP of any ecosystem and to analyse trends [42] and seasonal variations [43]. These analyses are made possible by the acquisition of satellite data with high temporal resolution over a long period, which can be used to quantify changes in ecosystems due to the internal dynamics of ecosystems and different climatic conditions [42].

Paruelo *et al.* [44] monitored ANPP from NOAA-AVHRR images in the Argentinean grasslands and verified that ANPP has a great spatial and interannual variability and that precipitation exerts a strong influence on ANPP. Fabricante *et al.* [45] monitored the ANPP in the grasslands of northern Patagonia (Argentina) by using data from the NOAA-AVHRR sensor correlated with rainfall data from weather stations. These authors found that the interannual variations of ANPP strongly depend on rainfall and that the correlation between ANPP and rainfall during the previous growing season was strong and the correlation between ANPP and rainfall in the current season was weak. Fonseca *et al.* [46] showed that it was possible to estimate ANPP in a Brazilian Pampa biome by modelling remote sensing and climate data and using spectral variables calculated from Landsat ETM+ images. In the areas of the Pampa biome in Brazil and Uruguay, Wagner *et al.* [47] observed negative trends in the MODIS-NDVI and MODIS-EVI (Enhanced Vegetation Index) time series during 2000 to 2011, which were associated with overgrazing and the occurrence of water deficits in shallow soils. Using the NOAA-AVHRR NDVI data in areas of the Pampa biome in Brazil between 1981 and 2000, Jacóbsen *et al.* [48] observed that the NDVI trends are similar to those of solar radiation availability and temperature, which both presented maximum values in the summer and minimum values in the winter. During the summer months of December and January, the NDVI values were reduced, likely because rainfall does not cover the demands of evaporation to the atmosphere and grazing in these areas.

Data have been obtained continuously from vegetation sensors since April 1998. The vegetation sensors have a spatial resolution of 1000 m, a daily temporal resolution and a swath width of 2250 km. Vegetation 1 and 2 sensors are on board the SPOT 4 (Satellite Pour l'Observation de la Terre) and SPOT 5 satellites, respectively. The images obtained from the vegetation sensors can be used to globally monitor the entire continental biosphere and provide images in the blue (0.43 to 0.47 μm), red (0.61 to 0.68 μm), near-infrared (0.78 to 0.89 μm), and shortwave infrared (1.58 to 1.75 μm) bands during periods of late morning sun [49]. The images of the bands and the NDVI are provided in the form of preprocessed products with radiometric (absolute calibration accuracy of approximately 5%) and geometric (less than 0.3 pixels) corrections and are distributed by the Vlaamse Instelling voor Technologisch Onderzoek (VITO). Data from the vegetation sensor are available in the following products: VGT-P, VGT-S1 and VGT-S10. The VGT-P product offers reflectance values from the top of the atmosphere and is used where physical data quality is important [49]. The VGT-S1 product uses the Simplified Model for Atmospheric Correction (SMAC) [50]. The VGT 10-S product is similar to the VGT-S1 product but includes global syntheses of ten days with daily observation derivatives selected using the maximum NDVI criterion [49].

Several studies have monitored the dynamics of ANPP using data from vegetation sensors. Xiao *et al.* [51] were able to characterize different forest types in Northeastern China when using the temporal variations of the NDVI and Normalized Difference Water Index (NDWI) calculated from this sensor imagery. In East Asia, Boles *et al.* [52] used vegetation sensors to classify land based on the temporal variations of the EVI and the Land Surface Water Index (LSWI). Lasaponara [53] used Principal Component Analysis (PCA) for a series of vegetation sensor images of Sicily to monitor landscape degradation in areas prone to desertification and strongly affected by forest fires. In addition, the African continent was analysed using data from the vegetation sensor. Tchuenté *et al.* [54] presented a hybrid clustering approach for classifying African ecosystems at a continental scale with an accuracy of 54% to 61% when compared with a reference map. To estimate forage availability in the Sahel, Jarlan *et al.* [55] used the NDVI from vegetation sensor data as an input for the STEP model (Sahelian Transpiration, Evaporation, and Productivity model) and obtained a correlation coefficient of 0.8 (n = 126) between the simulated data and the field foraging data. Do *et al.* [56] showed that the variability of the NDVI in the Sahel depends on the type of land use and was unable to verify the hypothesis that the interannual NDVI variations have identical sensitivity to any soil type.

The aims of this paper were to evaluate the changes in the ANPP of grasslands in the Pampa biome from experimental plots, to identify changes the spectral responses of similar vegetation communities obtained from orbital remote sensing and to compare both datasets with variations in meteorological elements to validate the transition scales of these datasets. We analysed and compared meteorological data trends for the period 1970 to 2011, remote sensing data for the period 1998 to 2011, and the average annual performance of locally obtained ANPP and NDVI data obtained from remotely sensed images.

## 2. Materials and Methods

### 2.1. Study Area, Scales and Data

**Figure 2:**
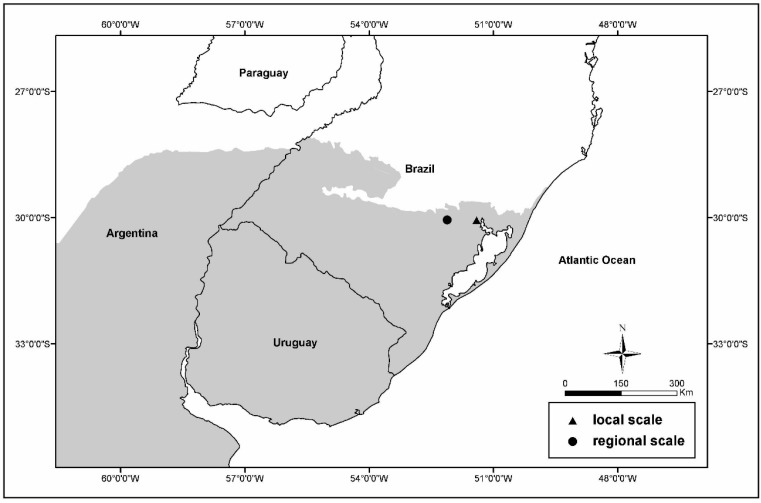
Positions of the areas analysed in the Pampa biome (represented in grey) at different scales. The regional scale areas (circle) analysed using the vegetation sensor and the local scale areas (triangle) analysed using a field dataset are presented, as well as the location of a weather station.

This study was performed using two different geographic scales according to an available dataset. At the local scale, an analysis of the climate and its direct influences on a grassland ANPP was performed using data obtained during a long-term experiment conducted by the Universidade Federal do Rio Grande do Sul (UFRGS). At the regional scale, an analysis of the influences of climate on grassland reflectance patterns was performed using data detected using vegetation sensor imagery. For this analysis, private cattle farms were considered. Both study areas are located in the state of Rio Grande do Sul in Brazil. These two study areas are shown in Figure 2, which shows that they are located at similar latitudes and approximately 80 km from each other.

The topography of the area consists of low, undulated relief, with elevations ranging between 50 and 200 m [57]. The vegetation in this area is rural, with a predominance of grasslands [3] that are characteristic of the Pampa biome. According to the Köppen climate classification system, the climate is Cfa (humid subtropical with hot summers). During the period 1970 to 2000, the average air temperature was 19.2 °C, with the coldest month having an average temperature of 13.5 °C (July) and the hottest month having an average temperature of 24.6 °C (January). The average rainfall at the study site is 1446 mm per year. The rainfall is well distributed throughout the year, with June being the wettest month (168.2 mm) and December being the driest month (97.7 mm). Solar radiation is the highest in December, with an average solar radiation of 509 cal·cm^−2^·month^−1^. The lowest amount of solar radiation occurs during June, and the average duration throughout the year is 206 cal·cm^−2^·month^−1^ [58].

#### 2.1.1. Description of the Long-Term Experiment 

A long-term experiment for “studies of the Pampa biome vegetation and its use as forage for livestock” has been conducted since 1986 in the Agronomic Experimental Station by the Department of Forage Plants, Faculty of Agronomy, UFRGS (30°06′12″S, 51°40′55″W, see the Supplementary Material-Figure S1 and S2). Cattle were allowed to graze over an area of approximately 52 ha that was divided into 14 experimental plots to simulate the management practices used by rural producers, which are known as gauchos in the Pampa biome. The botanical composition of the experimental area was described by Boldrini *et al.* [59]. The experimental plots were continuously subjected to grazing by beef cattle and separated according to the level of forage allowance (FA) offered to the animals [60], with two replicates for each type of management. The FA and zootechnical measures indicated a relationship between the amount of forage (kg·ha^−1^) and the live animal weight (kg) per unit area (ha), which provided an instantaneous measurement of the availability of forage for animals [61]. In the eight experimental plots, the forage allowances of 4%, 8%, 12% and 16% were held constant throughout the year. In the six experimental plots, the FA was adjusted during the annual seasons to simulate the same adjustments made by the farmers. Thus, the FA during the warm season (spring and summer) will be replaced by another FA during the cold season (autumn and winter), with variations from 8% to 12%, 12% to 8% and 16% to 12%. In this case, the first number is related to the warm season FA while the second number is related to the cold season forage allowance. ANPP measurements and the weights of grazing animals were determined monthly, which allowed for adjustments of the FA. No other interventions, such as fertilization, irrigation, cut or fire were made in the experimental plots [60], simulating the management performed by traditional producers in the region.

The ANPP dataset used in this study covered the period from October 2000 until July 2011, and data were collected on a monthly basis. Gaps were found in the dataset (of 129 months) due to a lack of field measurements; therefore, only 63 months were used. Despite the existence of older data collected during the same experiment, older data were not used because only four field measurements were made each year (one measurement during each season). The estimated ANPP (kg·ha^−1^ of aboveground dry matter) in each experimental plot was obtained using the technique described by Wilm *et al.* [62]. The monthly ANPP was estimated using four grazing exclusion cages of 1.5 × 1.5 m per experimental plot, which would enable the measurement of this variable without interference from the consumption of animals. Four sampling points were cut for each experimental plot. The ANPPs at each sampling point were collected in paper bags and dried in ovens at 65 °C for 72 h before weighing using a precision scale. The ANPP in each experimental unit was calculated by averaging the measurements made from the four cages, and the two sample plot replicates were averaged to obtain the ANPPs for each level of forage allowance [63].

#### 2.1.2. Selection and Identification of Monitored Areas at the Regional Scale

To select satellite monitored areas, two fieldwork experiments were performed on 16 December 2011 and 29 June 2012. Land use and size were the criteria used for selecting the areas. Six areas used for livestock grazing were selected with a spatial resolution higher than the vegetation sensor (1 km) (see on the complementary material). The geographic coordinates of the centre of the selected areas were obtained using a GPS receiver and by using WGS84 as the datum and latlong as a projection system. The geographic coordinates were used to identify six areas in the Google Earth Pro [64] software from the Landsat 5 satellite images available in its database. The limits of the selected areas were vectorized using the Google Earth Pro software, which was configured using the same geodetic parameters of the GPS receiver. Inside of these areas, the geographic coordinates of the centres of the vegetation pixels were used to vectorize the limits of the monitored areas and form a 1 km square. This process was used to identify a single pixel of the image from the vegetation sensor while retaining the surrounding area. The vegetation images were provided in the form of preprocessed products with a geometric correction (less than 0.3 pixels) by using WGS84 as the datum and latlong as the projection system (VITO). A visual analysis in a time series of Landsat/TM imagery was performed to identify possible land use changes in the six areas monitored during the period analysed in this study. For this analysis, 62 scenes (Path/Row 222/82) that were free of clouds between September 1997 and November 2011 were used, and no land use changes were verified in these areas.

Data from the vegetation sensor were extracted using the VGT S-10 product for the period from April 1998 to December 2011, for a total of 495 scenes during this time series (three files with 10 days of synthesis for each of the 165 months). Using the VGTExtract software, version 1.4.1, each file was converted from HDF images to TIFF images while retaining the original geodetic parameters. These images were analysed using the ENVI software, version 4.5. The “layer stacking” command was used, which considers a file containing the 495 scenes used in this study and the “region of interest” command, which consists of the contour vectors for each of the six monitored areas. The reflectance values in the red, near infrared, and shortwave infrared bands and the NDVI were extracted. These data were exported to a spreadsheet in which the averages of the six areas for each spectral band and the NDVI were performed comprising four time series.

#### 2.1.3. Meteorological Data

A weather station is located inside the Agronomic Experimental Station at UFRGS that has collected daily observations since 1970. Currently, the automatic weather station records weather data every 15 min. Global solar radiation, air temperature (minimum, average and maximum), precipitation, relative humidity, wind speed, evaporation and actual evapotranspiration (Penman) [58] data are collected. To analyse the climate, we used monthly average air temperature (minimum, average and maximum), total rainfall and total actual evapotranspiration data for the period 1970 to 2011. 

### 2.2. Data Analysis

#### 2.2.1. Climate Characterization and Trends

To evaluate the seasonal patterns of the meteorological dataset and characterize the climate in the study area, the average monthly minimum, mean and maximum air temperatures (°C), total rainfall (mm) and total actual evapotranspiration (mm) for the period 1970 to 2011 (42 years) were collected.

To observe trends in the meteorological dataset, we used the Mann-Kendall (MK) test. Which is a non-parametric test that does not require normally distributed data for validity [65]. This test is widely used in studies of hydrological, climatological and NDVI time series [65,66,67,68], where the Kendall rank correlation coefficient (τ) ranges from −1 to +1, and the statistical significance is tested using the *p*-value. For the meteorological dataset, the minimum level of significance that was adopted was α = 0.01. These analyses were performed using the full dataset and data separated into warm (September to April) and cold seasons (May-August). Grouping the months into warm and cold seasons was based on variations in the primary production presented by the Pampa biome, which was lower in the late fall and winter due to the lower air temperatures and frost and decreased the efficiency of the conversion of solar radiation in plant biomass [69,70]. To quantify trends in the meteorological dataset, we fit a linear regression model by using the least squares method. The selected independent variable was time (months), and the dependent variable was the meteorological variable.

#### 2.2.2. Spectral Reflectance Patterns for the Pampa Grasslands and Its Trends

To evaluate the Pampa spectral reflectance patterns from the vegetation sensor for each spectral band and the NDVI time series, we used a procedure that was similar to that used for the meteorological dataset. First, a linear regression model was fit using the least squares method to quantify the trends and the statistical significance of the resulting trends was verified using the Mann-Kendall test. Similarly, for the meteorological dataset, the selected independent variable was time (decendials), and the dependent variable consisted of each spectral band and the NDVI. The minimum level of significance adopted was α = 0.1. This level was used because of the large heterogeneity of the species in areas of the Pampa biome according to the results of Fonseca *et al.* [46]. The NDVI seasonal patterns were evaluated monthly using the average and standard deviation values for each ten-day synthesis (or decendial synthesis) and were calculated for the 1998 to 2011 NDVI time series. The seasonal NDVI pattern was analysed in association with the meteorological data and by considering the grassland management used by farmers. Pearson’s correlation analysis was performed between the NDVI and climate data using decendial periodicity. Lags of six 10-day periods were made to identify the peaks of these variables with time.

#### 2.2.3. Aboveground Seasonal Patterns of Net Primary Productivity

The monthly mean, maximum and minimum values of ANPPs for each forage allowance (4%, 8%, 12%, 16%, 8% to 12%, 12% to 8% and 16% to 12%) over 10 years (2001 to 2011) were used to evaluate the seasonal patterns of ANPP.

#### 2.2.4. Scale Analysis

At the local scale, the meteorological data were used to explain the seasonal variations of ANPP, and at the regional scale, the meteorological data were used to explain the seasonal variations of the spectral patterns detected in the satellite images. The local scale and regional scale datasets were both under the influence of the same climate. This influence was used to compare the ANNP and NDVI datasets acquired at the different spatial scales and indicated that the NDVI obtained from vegetation sensor imagery can be used as an indicator of ANPP at the regional scale [35,56,71,72]. No statistical relationships were fit between the NDVI and ANPP data because these data were collected in different areas and at different scales. However, a correlation analysis of the monthly ANNP and NDVI mean values was performed to compare the local and regional scale data. Because these data do not have the same periodicity, the NDVI average of each month was calculated to adjust the frequency of the data. The NDVI was compared with every level of forage allowance.

## 3. Results and Discussion

### 3.1. Climate Characterization and Analysis

**Figure 3:**
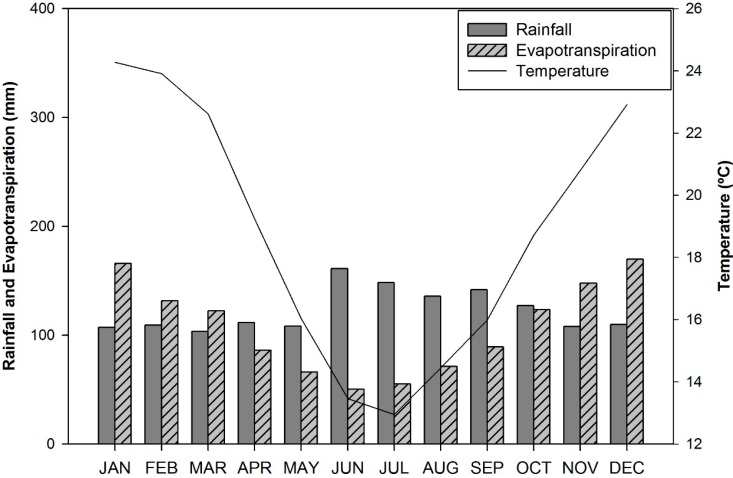
Seasonal patterns of the monthly mean air temperature, monthly total rainfall and actual evapotranspiration for the period 1970 to 2011.

Figure 3 shows the seasonal patterns of the monthly mean air temperature, the monthly total rainfall, and the actual evapotranspiration for the period 1970 to 2011. The months of January and July had the highest and lowest recorded temperatures, respectively, which were similar to those shown for the entire state of Rio Grande do Sul [73,74]. The rainfall regime in the study area followed a regular precipitation distribution among the four seasons, which was similar to the results observed by Viana *et al.* [75] for this region.

#### 3.1.1. Trend Analysis of Rainfall and Actual Evapotranspiration

**Figure 4:**
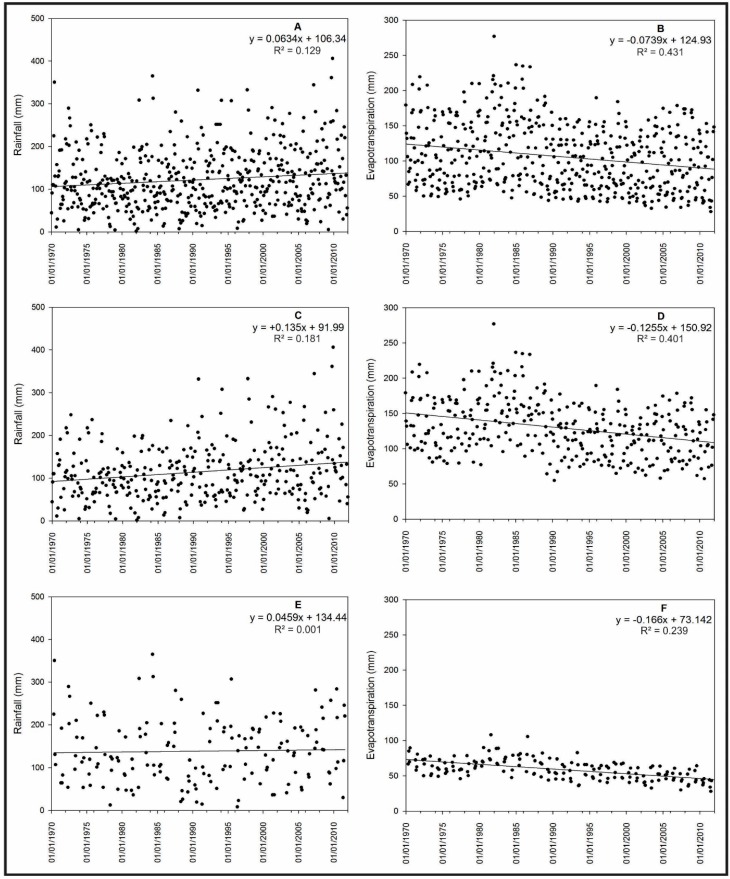
Trends of the meteorological variables during the time series, including the total rainfall (**A**); total actual evapotranspiration (**B**); total rainfall in the warm season (**C**); total actual evapotranspiration in the warm season (**D**); total rainfall in the cold season (**E**); and total actual evapotranspiration in the cold season (**F**).

Figure 4 shows the precipitation and evapotranspiration trends with the adjusted linear model equation and R^2^ values. The R^2^ is a measure of the amount of variation in a variable that is explained by another variable and is calculated using the square root of Pearson’s correlation coefficient. A positive trend of 31.95 mm of total rainfall during the 41 analysed years was observed in the total monthly rainfall from the full dataset collected between 1970 and 2011. This trend was statistically significant according to the Mann-Kendall test (*p*-value = 0.003) (Figure 4A) and was similar to the results obtained by Barros *et al.* [76], who observed an increase in total rainfall in an area of southern Brazil located approximately 100 km from the area analysed in this study during the period 1960 to 1999. For the actual monthly evapotranspiration, a reducing trend of −37.2 mm during the 41 analysed years was observed that was statistically significant according to the Mann-Kendall test (*p*-value = 0.0001) (Figure 4B). When analysed together the rainfall and actual evapotranspiration could be used to make inferences regarding soil moisture [77] and the availability of water to plants. Although the actual evapotranspiration generally decreased and the amount of rainfall generally increased, we were able to observe a trend of increasing water availability in the soil during the analysed years. In the warm season, a similar behaviour was observed, which indicated a trend of increasing soil water availability. A positive trend in monthly total rainfall was found with an increase of 45.36 mm between 1970 and 2011 (*p*-value = 0.003, for Mann-Kendall test) (Figure 4C). Regarding the actual monthly evapotranspiration, a negative trend was observed that represented a reduction of 42.1 mm during the study period (*p*-value = 0.0001, for Mann-Kendall test) (Figure 4D). For the cold months, higher soil water availability was observed during the analysed period because a negative trend of −27.8 mm was observed for the monthly actual evapotranspiration (statistically significant according to the Mann-Kendall test with a *p*-value = 0.0001) (Figure 4F), although the trend for rainfall was not significant (Figure 4E).

#### 3.1.2. Analysis of Air Temperature Trends

**Figure 5:**
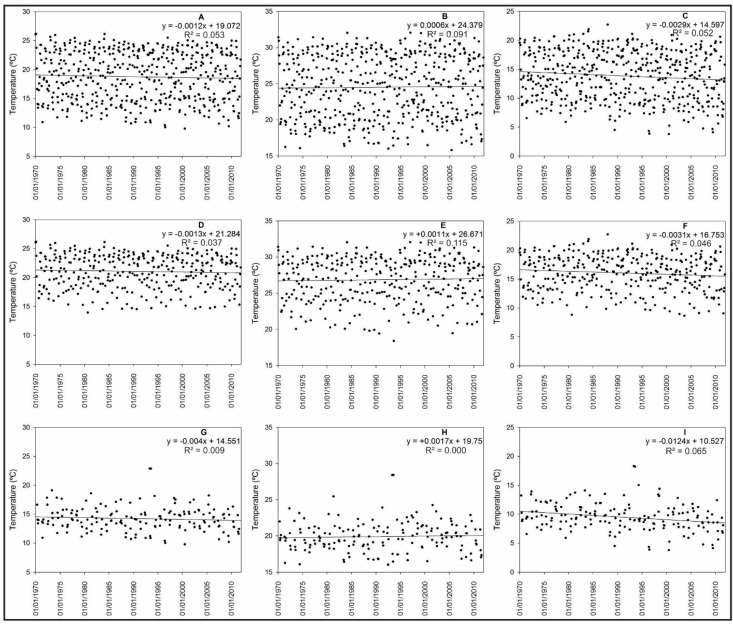
Trends for the time series of the meteorological variables, including the average air temperature (**A**); maximum air temperature (**B**); minimum air temperature (**C**); average air temperature in the warm season (**D**); maximum air temperature in the warm season (**E**); minimum air temperature in the warm season (**F**); average air temperature in the cold season (**G**); maximum air temperature in the cold season (**H**); and minimum air temperature in the cold season (**I**).

Figure 5 shows the temperatures trends, the adjusted linear model equation and the R^2^ values. The monthly average air temperatures (T_avg_) and monthly minimum air temperature (T_min_) (Figure 5A,C) presented decreasing trends that were statistically significant according to the Mann-Kendall test. The generated linear models indicated a decreasing trend for a T_min_ of −1.46 °C and for a T_avg_ of −0.49 °C in 41 years. No trend was observed for the monthly maximum air temperatures (T_max_) (Figure 5B). This result is different from the trends observed in other studies that analysed a time series of temperature in southern Brazil, which presented an upward trend in the minimum air temperature [67,68,78] when considering a different number of years. However, when analysing the warm and cold season datasets separately, these authors found that the results did not demonstrate an increasing trend in air temperatures. During the warm season, the trends were not significant for any of the three analysed variables (Figure 5D–F), which indicated a stable trend in the air temperatures during the warmer months of the year. During the cold season, the trend was only statistically significant for T_min_, which exhibited a slope of −0.0124 and a negative trend of −2.08 °C over the 41 analysed years (Figure 5I).

**Figure 6:**
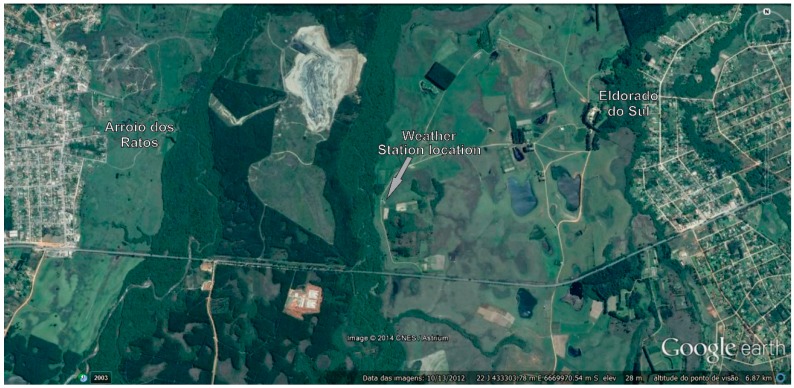
Position of the weather station and the urban areas near the experimental plots.

The negative or neutral trends for the air temperature variables can be attributed to the greater availability of soil water, which allows for a more dynamic energy balance process [79]. Other causes may include the location of the weather station, which is located 2.5 km from the town of Eldorado do Sul (37,366 habitants) and 3.7 km from Arroio dos Ratos (13,606 habitants) (Figure 6), and the absence of land use and land cover changes over the 41 analysed years near the weather station. 

### 3.2. Monthly Evaluation of ANPP Data at the Local Scale

**Figure 7:**
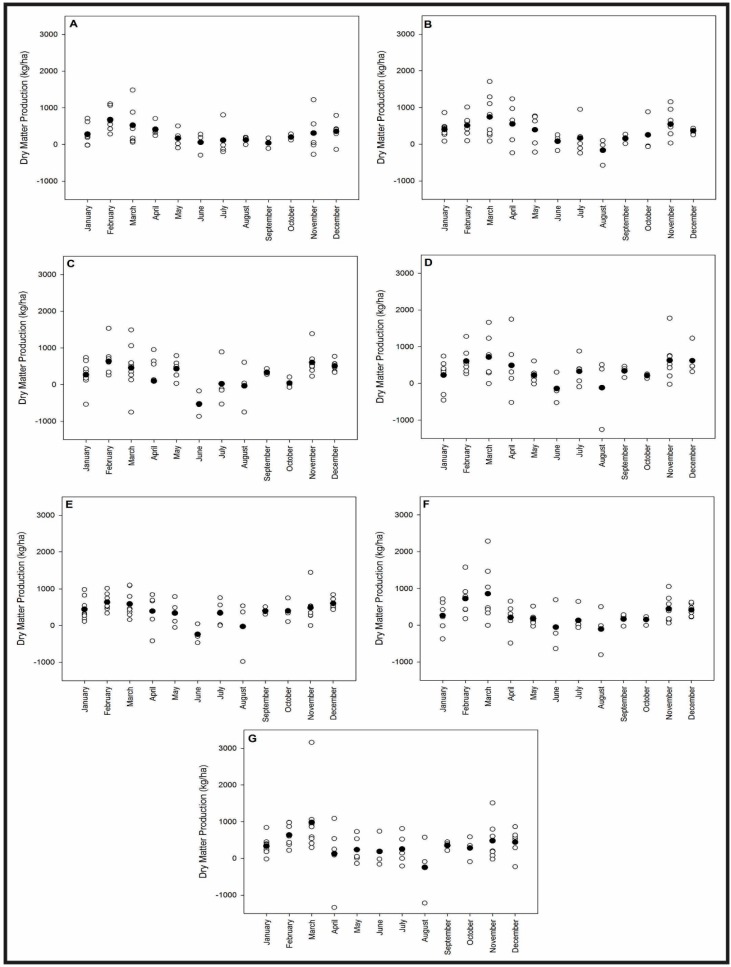
Average monthly pattern (black dots) and dispersion (white dots) for the ANPPs for forage allowance: 4% (**A**); 8% (**B**); 12% (**C**); 16% (**D**); 8 to 12% (**E**); 12 to 8% (**F**); and 16 to 12% (**G**).

The monthly mean ANPP values for each forage allowance for 10 years (2001 to 2011) and the monthly data dispersion for the same years are presented in Figure 7. The mean ANPP values increased as the spring season began (September) and reached their maximum values during the late summer and at the beginning of the autumn season (February–March) for all forage allowances, which coincided with the main growth season of the Pampa grasslands [12,80,81,82,83]. During the autumn and winter seasons, the mean ANPP values decreased for all forage allowances. These results can be explained by the predominance of C_4_ species relative to C_3_ species in this biome [4,81,82]. C_4_ species have higher growth rates during the warm season, and C_3_ species have more efficient photosynthesis processes in cold weather [84,85]. For all forage allowances, the mean ANPP values were higher in July than in June due to the growth of the C_3_ species. The ANPP values for March exhibited a greater range than the other monthly data and showed greater interannual variability at the end of the warm season. Thus, at the local scale, the data from ANPP corresponded with the availability of solar radiation and air temperature, with a maximum ANPP occurring during the summer and a minimum value occurring during the winter. Bettolli *et al.* observed similar results for grasslands in the Pampa biome [12]. These authors showed that ANPP values were maximized during the summer and minimized during the winter for three soil types from 1980 to 1994 in the region of Salto, Uruguay. The same pattern of ANPP was recorded in Patagonia grasslands using field measurements [86,87].

### 3.3. Variations of the Spectral Reflectance of Grasslands at the Regional Scale and Their Relationships with Meteorological and ANPP Datasets

**Figure 8:**
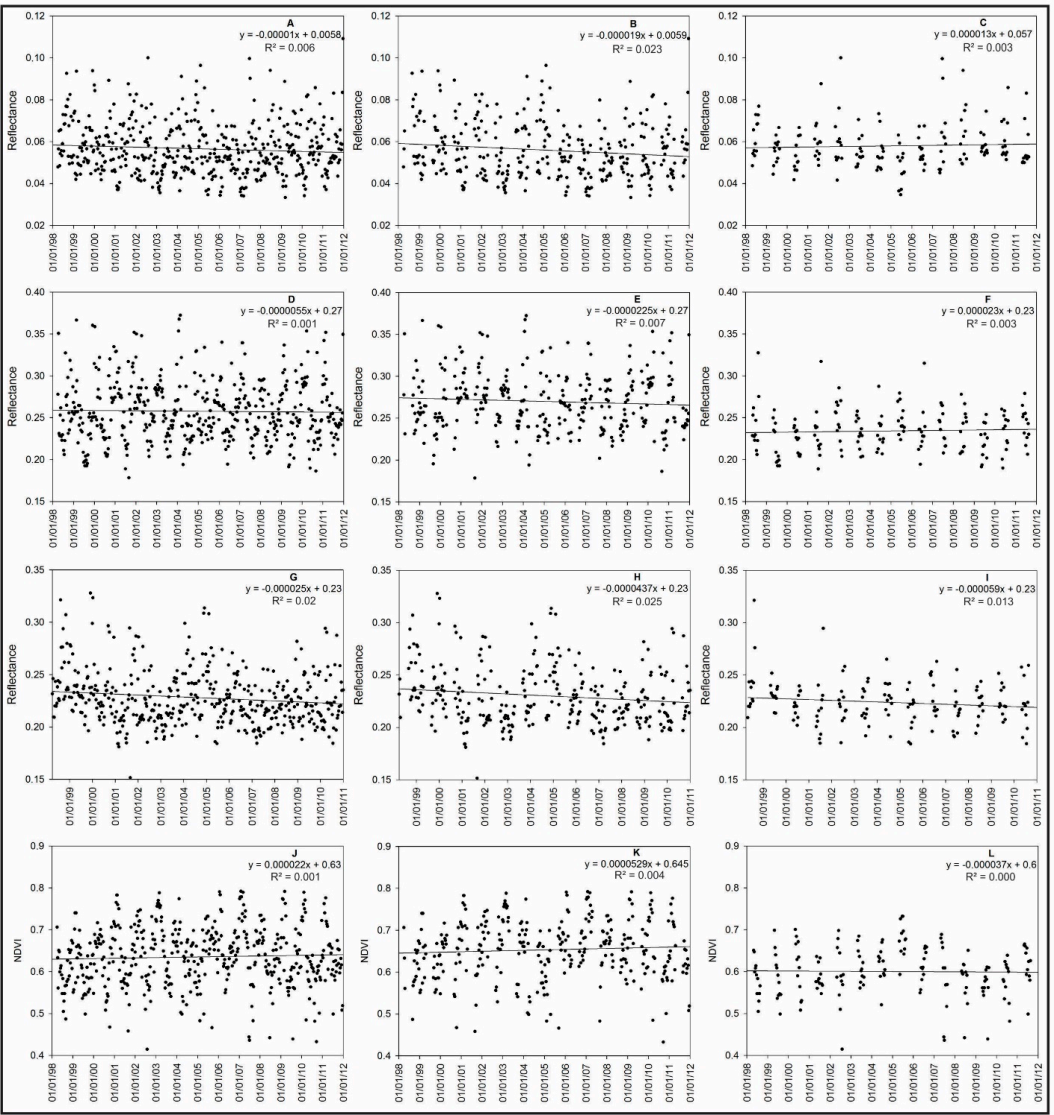
Trends for the spectral time series variables, including the red band (**A**); red band in the warm season (**B**); red band in the cold season (**C**); near-infrared band (**D**); near-infrared band in the warm season (**E**); near-infrared band in the cold season (**F**); shortwave infrared band (**G**); shortwave infrared band in the warm season (**H**); shortwave infrared band the in cold season (**I**); NDVI (**J**); NDVI in the warm season (**K**); and NDVI in the cold season (**L**).

To analyse the spectral variations in the Pampa biome grasslands relative to the ANPP seasonal variations, the results obtained from the trend analysis of spectral variables were compared with the results obtained for the meteorological variables because the weather is responsible for variations in the ANPP in the Pampa biome [43,44,48]. In addition, the results were compared with the variations in the ANPP values that were collected at the local scale. The trends for the entire dataset for the warm and cold season datasets are shown in Figure 8.

For the warm season, the reflectance of the red wavelength showed a significantly negative trend, and no trends were observed for the cold season or for the entire period. A reduction in the canopy reflectance in this wavelength is associated with an increase in the leaf area index (LAI) due to an increase in the ANPP [33,88,89,90,91,92]. The local scale data show that the main growth season of the Pampa biome was during the spring, summer and beginning of autumn, and that only small amounts of growth occurred during the cool season (Figure 7). During the warm season, the ANPP values were greater at the end of the season, which explained the negative trend in the tendency analysis. During the cold season, the small growth in the Pampa grasslands is very difficult to detect by using satellite imagery because all of the biomass production is consumed by cattle in a situation called overgrazing, which is typical in this biome [8,93] and occurs when the consumption of biomass by animals is greater than the ANPP of the vegetation that is used to feed them. When compared with meteorological data trends, the significant negative trends observed in the red wavelength are related to increases in the amount of water available for vegetation, which optimizes the photosynthesis process and results in an increase in the radiation absorption and a decrease in the reflectance [94,95].

For the shortwave infrared reflectance a significant negative trend (*p*-value = 0.002) was observed for the complete dataset and the warm season (*p*-value = 0.010). No trends were observed for the cold season. These results were also related to increases in the available water for vegetation because the reflectance at this wavelength decreases when the canopy water status increases [27,31] and the reflectance of the water absorption bands increases (1.4; 1.9; 2.7 μm) [19]. When these patterns of reflectance are analysed using shortwave infrared wavelengths and their relationships with the meteorological data, we can confirm that the water available for the vegetation in this region increases (Brazilian southern) and that the Pampa grassland species use this water for photosynthesis processes.

No trends were observed in the near-infrared reflectance and the NDVI, which indicated the stabilities of these variables along the time series and showed that no significant interannual changes were detected in the productivity of the analysed areas between April 1998 and December 2011 when using imagery with a low spatial resolution. The NDVI is correlated with the biophysical properties of vegetation cover; thus, no significant changes were detected in the LAI or biomass [41] between April 1998 and December 2011. In other studies that analysed the NDVI at large scales, Fensholt and Proud [96] observed a decrease in the ANPP during the period 2000 to 2010 when considering the NDVI time series from the MODIS sensor in northeastern Argentina, Paraguay, Uruguay and west of the state of Rio Grande do Sul state in Brazil. However, the central areas in the state of Rio Grande do Sul presented a stable NDVI. A similar result was observed by Zhao and Running [97] in the central area of the state of Rio Grande do Sul when using a series of data obtained from a MODIS sensor for the period 2000–2009. The results of this study confirm the stability of ANPP in the central area of the state of Rio Grande do Sul with a time series from the vegetation sensor for the period between April 1998 and December 2011.

**Figure 9:**
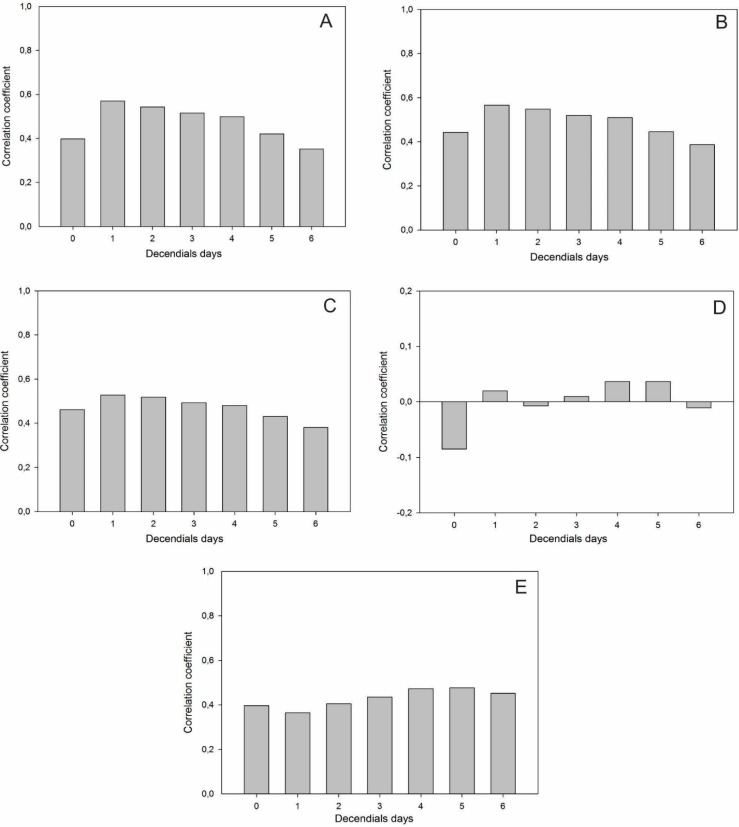
Correlation between NDVI and climate variables: (**A**) NDVI *versus* minimum air temperature; (**B**) NDVI *versus* average air temperature; (**C**) NDVI *versus* maximum air temperature; (**D**) NDVI *versus* total rainfall; (**E**) NDVI *versus* evapotranspiration. The numbers on the horizontal axis represent the time lags in decendials days.

Figure 9 shows Pearson’s correlation between the NDVI and the meteorological variables with six decendial lags. A positive and statistically significant correlation with a probability of 99% between the NDVI and temperature values was observed for all lags. The highest values occurred with a lag of one decendial above 0.5, with a reduction in the coefficient by increasing the lag. The correlation of NDVI with precipitation did not show any pattern and oscillated between positive and negative values with a low correlation and with no statistical significance. In northern Patagonia (Argentina), Fabricante *et al.* [45] found a strong correlation between ANPP and the amount of rainfall that occurred during the previous growing season. For evapotranspiration, the values ranged from 0.364 to 0.476 mm, and the strongest correlation was shown for a lag of five decendials days. 

### 3.4. Evaluation of NDVI Seasonal Patterns at the Regional Scale

**Figure 10:**
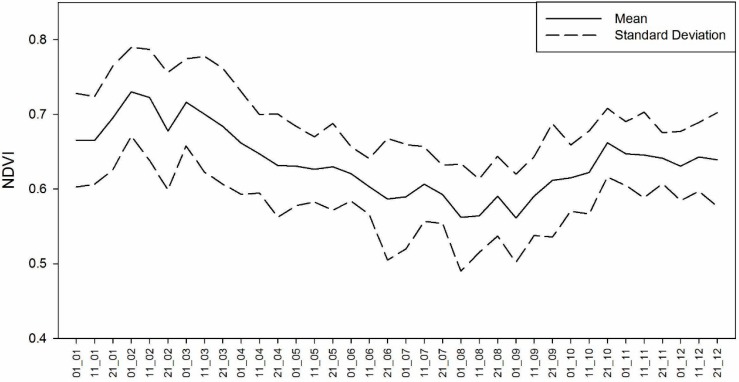
The yearly pattern derived by averaging the same-date over 13 years. Dispersion values are also represented. The values on the x-axis represent the decendial synthesis (1, 11, 21) and the month of the year (1 to 12).

The NDVI values were analysed monthly using the average, maximum and minimum values for each ten-day synthesis (or decendial synthesis) calculated over the 1998 to 2011 NDVI time series. Figure 10 shows the NDVI seasonal pattern, in which a large range of values for each ten-day synthesis can be observed, with standard deviation values ranging from −0.1 to +0.1. This behaviour is typical and results from the interannual variability of air temperature and precipitation, as shown in Figure 4 and Figure 5. These variables condition the biomass accumulation process; therefore, once they have a strong interannual variation, the same pattern is expected for the NDVI.

The average variability of the NDVI between the decendials results from the variability of the meteorological data, especially the air temperature (Figure 4), and behaves similarly to the monthly mean ANPP (Figure 7). The average NDVI increases when the spring season begins (September) and reaches its maximum value in the late summer and toward the beginning of autumn (February–March). In November and December, the NDVI average values are stabilized at approximately 0.65, despite the increase in ANPP (Figure 7) during this same period that was observed in most of the forage allowances. This stability potentially resulted from grazing because these analyses were made in grazing areas. Nabinger, Moraes and Maraschin [98] showed that higher rates of daily weight gains for cattle occurred from September to February, the period of main vegetation growth in the Pampa biome. These variations in the NDVI are potentially associated with a reduction in the amount of water available for vegetation because the evapotranspiration is greater than precipitation from November to March (Figure 3). Using the AVHRR sensor to study the temporal and spatial variability of the Pampa biome, Fontana *et al.* [99] and Jacóbsen *et al.* [48] observed a reduction in the NDVI values that was associated with a reduction in the amount of water available for vegetation. 

### 3.5. Comparison of Data at the Regional Scale 

Table 1 presents the linear correlation coefficients for the mean monthly NDVI values for each forage allowance. These correlations show strong linear relationships between the NDVI and the forage allowance and actual aerial biomass in the Pampa biome grasslands, which are statistically significant at 99% or 95%. The highest correlation occurred for an NDVI of 4%, which exhibited a correlation coefficient of 0.9 and decreasing forage allowances of 8% to 12%, 8%, 16% to 12%, 8% to 12%, 16%, and 12%.

**Figure 11:**
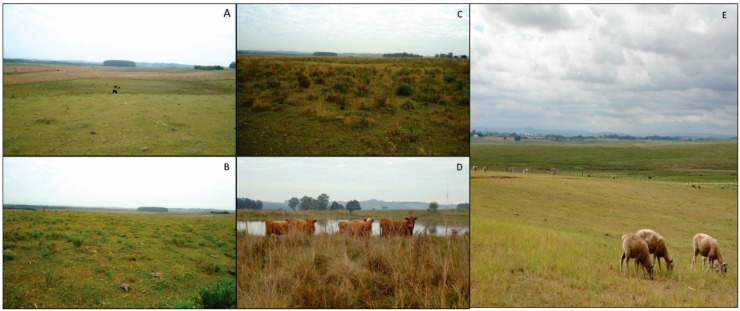
Landscapes of the experimental plots for 4% (**A**); 8% (**B**); 12% (**C**); 16% (**D**); forage allowance and for the monitored areas at the regional scale (**E**).

Although the forage allowances of the grasslands in the monitored areas at the regional scale have not been evaluated (Figure 11E), they are presumed to be approximately 4% (Figure 11A) or 8% (Figure 11B) because the forage allowance is commonly 4% in this region due to the overgrazing practices used by local farmers [8]. Thus, despite a significant correlation between the NDVI and forage allowance, the highest correlation between the NDVI and a forage allowance of 4% is consistent with the management practices used in the monitored areas at the regional scale.

The ANPP dataset collected at the local scale excludes animal grazing through the use of exclusion cages but does not measure the production of species in the Pampa biome that animals do not eat. The NDVI dataset collected at the regional scale by using vegetation sensors includes all species present in the area but the quality of the data is affected by several problems related to the acquisition of remote sensing data, including saturation of the sensor, the illumination geometry, shading and soil [100,101,102]. Although these two datasets have different spatial scales, these results show that the monthly variations of the growth of vegetation canopies analysed with environmental changes (temperature, total rainfall and total evapotranspiration) were similar. Therefore, data at the local scale can be expanded to a regional scale in the Pampa biome.

**Table 1:** Correlation coefficient between the NDVI and forage allowance.

Forage Allowance	NDVI
4%	0.90 **
8%	0.86 **
12%	0.58 *
16%	0.67 *
8%–12%	0.68 *
12%–8%	0.77 **
16%–12%	0.87 **

* statistical significance for α = 0.05; ** statistical significance for α = 0.01.

## 4. Conclusions

Some important and statistically significant trends of changes in the values of meteorological values for the period between 1970 and 2011 were observed, including an increase of 31.95 mm in the total rainfall, a decrease of 37.2 mm in the total actual evapotranspiration and a decrease of 1.46 °C in the minimum air temperature for the study area. These trends indicated an increase in plant available water, due to the positive trend in rainfall and the negative trend in actual evapotranspiration.

From the analysis of the remote sensing dataset for the period between April 1998 and December 2011, standard patterns were observed for the reflectance values in red and the shortwave infrared spectral bands. No trends within the near-infrared reflectance and the NDVI time series were found, and because these variables directly influenced the green biomass of the vegetation, the cause of stability can be explained by the consumption of cattle foraging in areas of the Pampa biome.

The results from this analysis of aboveground net primary production data at the local scale and the NDVI data from the vegetation sensor at the regional scale showed that variations in the grassland growth were similar and independent of the scale of analysis, which indicated that data collected at the local scale and its relationships with climate can be expanded at the regional scale in the Pampa biome by using remote sensing techniques.

## Supplementary Materials

Supplementary materials can be accessed at: http://www.mdpi.com/1424-8220/15/7/17666/s1.
