# Supplementary material for: Multiscale Trend Analysis for Pampa Grasslands Using Ground Data and Vegetation Sensor Imagery
Source: Sensors (Basel). 2015 Jul 21;15(7):17666–92. doi: 10.3390/s150717666 (PMC4541953; doi:10.3390/s150717666)
Supplement: Supplementary file 1 [file sensors-15-17666-s001.pdf]

OPEN ACCESS

sensors

ISSN 1424-8220

www.mdpi.com/journal/sensors

Supplementary Information

Multiscale Trend Analysis for Pampa Grasslands Using Ground Data and Vegetation Sensor Imagery. *Sensors* 2015, *15*, 17666-17692

Fernando C. Scottá * and Eliana L. da Fonseca

Department of Geography, Federal University of Rio Grande do Sul, Porto Alegre 91501-970, RS, Brazil; E-Mail: eliana.fonseca@ufrgs.br

* Author to whom correspondence should be addressed; E-Mail: fernando.scotta@ufrgs.br; Tel.: +55-51-3308-6343.

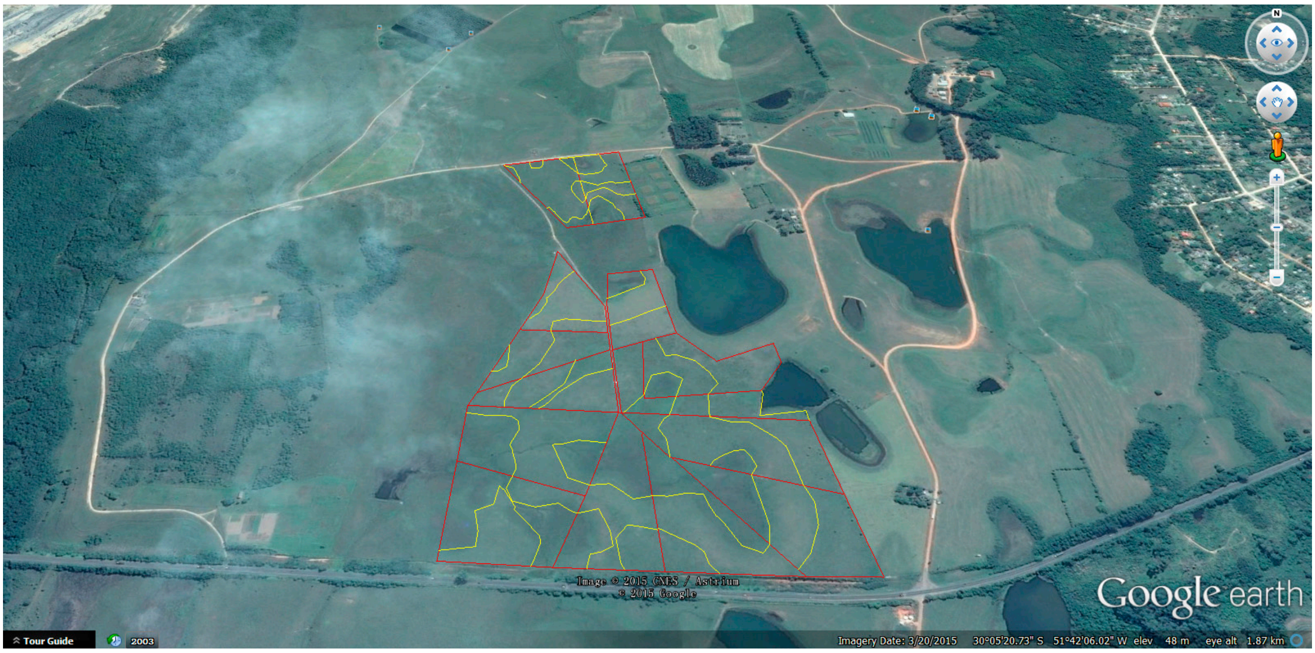

Figure S1. Local Scale Plots: location of long-term experiment where the ANPP dataset were collected.

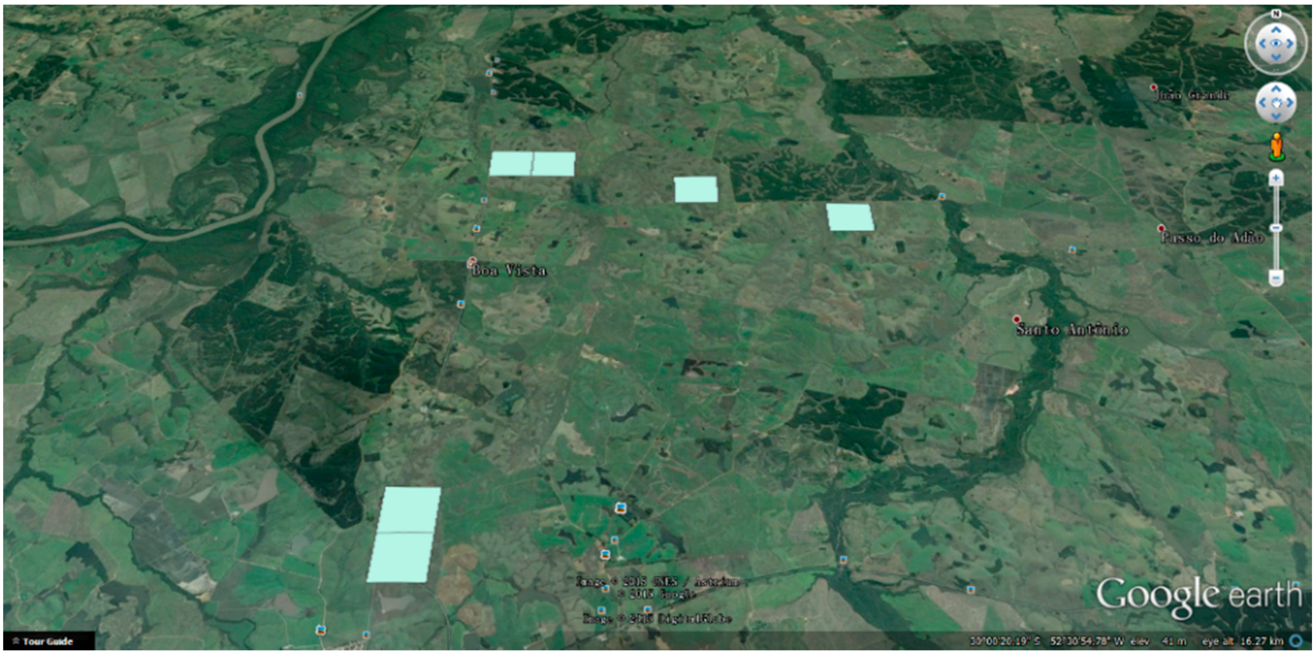

Figure S2. Regional Scale Plots: six areas used for monitoring at the regional scale.

© 2015 by the authors; licensee MDPI, Basel, Switzerland. This article is an open access article distributed under the terms and conditions of the Creative Commons Attribution license (http://creativecommons.org/licenses/by/4.0/).
